# Analysis of *Aspergillus nidulans *metabolism at the genome-scale

**DOI:** 10.1186/1471-2164-9-163

**Published:** 2008-04-11

**Authors:** Helga David, İlknur Ş Özçelik, Gerald Hofmann, Jens Nielsen

**Affiliations:** 1 Fluxome Sciences A/S, Diplomvej 378, Kgs. 2800 Lyngby, Denmark; 2 TÜBİTAK – The Scientific and Technological Research Council of Turkey, Tunus Caddesi No: 80 06100 Kavaklýdere, Ankara, Turkey; 3 Novozymes A/S, Fermentation Optimization, Hallas Alle 1, Building BD3.44, DK-4400, Kalundborg, Denmark; 4 Center for Microbial Biotechnology, BioCentrum-DTU, Building 223, Technical University of Denmark, DK-2800 Kgs. Lyngby, Denmark

## Abstract

**Background:**

*Aspergillus nidulans *is a member of a diverse group of filamentous fungi, sharing many of the properties of its close relatives with significance in the fields of medicine, agriculture and industry. Furthermore, *A. nidulans *has been a classical model organism for studies of development biology and gene regulation, and thus it has become one of the best-characterized filamentous fungi. It was the first *Aspergillus *species to have its genome sequenced, and automated gene prediction tools predicted 9,451 open reading frames (ORFs) in the genome, of which less than 10% were assigned a function.

**Results:**

In this work, we have manually assigned functions to 472 orphan genes in the metabolism of *A. nidulans*, by using a pathway-driven approach and by employing comparative genomics tools based on sequence similarity. The central metabolism of *A. nidulans*, as well as biosynthetic pathways of relevant secondary metabolites, was reconstructed based on detailed metabolic reconstructions available for *A. niger *and *Saccharomyces cerevisiae*, and information on the genetics, biochemistry and physiology of *A. nidulans*. Thereby, it was possible to identify metabolic functions without a gene associated, and to look for candidate ORFs in the genome of *A. nidulans *by comparing its sequence to sequences of well-characterized genes in other species encoding the function of interest. A classification system, based on defined criteria, was developed for evaluating and selecting the ORFs among the candidates, in an objective and systematic manner. The functional assignments served as a basis to develop a mathematical model, linking 666 genes (both previously and newly annotated) to metabolic roles. The model was used to simulate metabolic behavior and additionally to integrate, analyze and interpret large-scale gene expression data concerning a study on glucose repression, thereby providing a means of upgrading the information content of experimental data and getting further insight into this phenomenon in *A. nidulans*.

**Conclusion:**

We demonstrate how pathway modeling of *A. nidulans *can be used as an approach to improve the functional annotation of the genome of this organism. Furthermore we show how the metabolic model establishes functional links between genes, enabling the upgrade of the information content of transcriptome data.

## Background

*Aspergillus nidulans*, also known as *Emericella nidulans*, as it can undergo sexual reproduction in its life cycle in addition to the non-perfect (asexually reproducing) form that characterizes aspergilli, is an important member of the filamentous fungal genus *Aspergillus*. This genus encompasses a large diversity of species of great medical and economical relevance. In the medical and agricultural fields, *A. flavus *and *A. parasiticus *represent major producers of mycotoxins (e.g. aflatoxins) that can contaminate important food and feed crops, while *A. fumigatus *may cause serious diseases in immuno-compromised animals and humans (e.g. invasive pulmonary aspergillosis). From a biotechnological viewpoint, *Aspergillus *species represent important industrial producers of diverse products, such as industrial enzymes (e.g. amylases by *A. niger *and *A. oryzae*), bulk chemicals (e.g. citric acid by *A. niger*), and pharmaceuticals (e.g. lovastatin, a cholesterol lowering-agent, by *A. terreus*).

Whereas the first efforts made in fungal genome research have focused on yeasts, there has been an increasing focus on filamentous fungi due to their medical, agricultural and biotechnological importance. There are quite large differences between yeast and most filamentous fungal genomes, with the latter exhibiting larger genomes owing to larger centromers and lower gene density per nucleotide length as well as the presence of far more genes. Furthermore, many of the filamentous fungal genes have a more complex structure due to the presence of multiple introns [1]. *A. nidulans *has become one of the model organisms of choice for filamentous fungal genome research as it is a representative of the important group of aspergilli, but also because this fungus has served as a model organism for studies of cell development and gene regulation [2]. It is one of the most extensively studied organisms in the fields of genetics and biochemistry, and this is obviously of great value in the identification of the function of orphan filamentous fungal genes and characterization of the biological roles of their products.

Genome-sequencing projects of several *Aspergillus *species have recently been completed (*A. fumigatus*, *A. nidulans*, *A. niger*, *A. oryzae*, *A. parasiticus*) or are nearing completion (*A. flavus*, *A. terreus*) [3,4]. In particular, the genomic sequence of *A. nidulans *(strain FGSC A4) was released by the Broad Institute of MIT and Harvard, with a13-fold coverage, in Spring 2003 [5]. The size of its genome is approximately 31 Mb, and it is organized in 8 chromosomes. 9,541 open reading frames (ORFs) were predicted using automated gene prediction tools (FGENESH, FGENESH+, and GENEWISE), and PFAM (protein family) [6] matches were identified by Hmmer analysis. However, due to the highly conservative criteria adopted in the gene naming process, and also due to the relative low number of genes characterized before whole genome sequencing, more than 90% of all ORFs identified are called hypothetical or predicted proteins.

In order to improve the predictions and improve the annotation, automatically assigned genes should be subjected to manual curation. Herein, integration of different types of data and combination of diverse genomic tools play a major role. Functional assignments of genes based on genomic data may be complemented with information providing biochemical and physiological evidence. Of special importance in functional genomics are high-throughput data generated by post-genomic techniques (e.g. transcriptome data using hybridization arrays [7]), which provide genome-wide screens of gene function [3,8,9]. In addition to similarity-based tools (e.g. BLAST [10], FASTA [11]), a number of methods are available for comparative genomics that combine various types of genomic evidence, such as protein fusion events [12], gene clustering on the chromosome [13], occurrence profiles or signatures [14], shared regulatory sites [15], which enable the so-called "gene context analysis" [16].

Metabolic reconstructions can give a valuable contribution to functional genomics, by uncovering missing metabolic functions (i.e., functions not assigned to genes) and hence putting forward the identification of the corresponding genes [16,17]. The underlying idea is that larger functional systems (e.g. metabolic pathways) require the presence of their components or elementary functional units (e.g. enzymatic steps) in order to be operative. Hence, in this approach, efforts are directed towards discovering (uncharacterized) genes within the sequenced genome that have a defined role in the metabolism, in opposition to strategies that aim at predicting the functions of a given set of genes known to be present. Comparative genome analysis may then be accomplished with well-characterized genes of related organisms, by employing diverse tools of comparative genomics (based on sequence similarity and gene context), and a prioritized list of potential candidate genes for the function of interest generated. The functional assignment of genes may be verified eventually by using experimental techniques. This approach has been previously described by Osterman and Overbeek [16], and a computational method has been developed for automated large-scale predictions of protein function [18]. This framework has been applied to prokaryotes (*Thiobacillus ferrooxidans *[19]) and eukaryotes (human [20]), and here we used it for the annotation of metabolic genes within the genome of *A. nidulans*.

In particular, we focused on the functional annotation of the genes involved in the central metabolism of this fungus, as well as on the biosynthetic pathways of relevant secondary metabolites. For the purpose, we reconstructed the central metabolic network of *A. nidulans*, based on detailed metabolic reconstructions of other eukaryotes, namely *A. niger *[21], *Saccharomyces cerevisiae *[22], and *Mus musculus *[23]. In what concerns the secondary metabolism, pathways for the biosynthesis of penicillin in *Penicillium chrysogenum *[24] and aflatoxins in different species of *Aspergillus *[25] were used as templates. Pathway prediction was also supported by available information on the biochemistry and physiology of *A. nidulans*. Our analysis assigned functions to 472 orphan ORFs in the metabolism of *A. nidulans*, by employing similarity-based tools of comparative genomic analysis (BLAST) and using public (non-redundant) databases of genes and proteins of established function [26]. These functions represented either missing enzymatic activities, for which no ORFs had been previously identified, or previously assigned functions, for which additional isogenes were discovered. The functional assignments made for the individual ORFs were integrated into a mathematical model that can be used to simulate metabolic behavior, thereby enabling a comprehensive and integrative analysis of metabolic functions in *A. nidulans*. Furthermore, the information contained in the metabolic reconstruction can be exploited for the analysis of large-scale transcription data. To illustrate this, the reconstructed metabolic network of *A. nidulans *was used in connection with an algorithm developed by Patil and Nielsen [27] for the large-scale analysis of gene expression profiles, in particular for studying transcriptional responses to specific genetic changes in *A. nidulans *(deletion of the regulatory gene *creA*) [28]. In a previous study, the metabolic network reconstructed in this work was employed in the study of the effects of changes in the environmental conditions (carbon source) on the transcriptome profiles of *A. nidulans *[29].

## Results

### Metabolic reconstruction and identification of candidate ORFs

The pathways predicted to take part in the central metabolism of *A. nidulans*, as well as those involved in the biosynthesis of secondary metabolites of interest, are listed in Tables 1 and 2 and Additional file 1. Our analysis assigned metabolic roles to 472 ORFs within the genome of *A. nidulans *that had not been annotated earlier (Table 1). In total, 666 ORFs were associated to functions in the metabolic reconstruction, including 194 previously annotated ORFs in the *Aspergillus nidulans *Database [5]. However, the correlation between the ORFs and metabolic functions is rather complex, because about 92 ORFs were found to be constituents of enzyme complexes, more than 100 ORFs were considered to be multifunctional, and approximately 190 ORFs to encode various isoenzymes. The reconstructed metabolic network included 676 unique biochemical reactions (551 cytosolic, 103 mitochondrial, 5 glyoxysomal, and 17 extracellular) and 113 unique transport processes. The analysis did not include transporters, and hence the transport reactions considered were based on those existing in related organisms, based on previous annotations for *A. nidulans *or concerned diffusional processes. Moreover, Additional file 1 includes information on the common metabolic reactions to *A. nidulans *and *A. niger *or *S. cerevisiae *networks.

**Table 1:** Number of ORFs associated to the metabolic reactions in the metabolic reconstruction for *A. nidulans*.

**Part of metabolism**	**No. of previously annotated ORFs [5]**	**No. of newly annotated ORFs**	**Total no. of ORFs^1^**
**Biochemical reactions**	**188**	**468**	**656**
C-compound metabolism	96	166	262
Energy metabolism	14	40	54
Aminoacid metabolism	40	125	165
Nucleotide metabolism	10	44	54
Lipid metabolism	13	97	110
Secondary metabolism	16	14	30
Nitrogen and sulphur metabolism	2	3	5

**Transport processes**	**6**	**3**	**9**

**TOTAL**	**194**	**472**	**666**

^1 ^The total number of unique ORFs in the metabolic network may be different from the sum of the number of ORFs in the different parts of the metabolism, because there are ORFs which code functions in several parts of the metabolism.

**Table 2:** Total number of biochemical conversions and transport processes in the reconstructed metabolic network of *A. nidulans *and comparison with the metabolic networks of *A. niger *(316 unique reactions) and *S. cerevisiae *(843 unique reactions).

**Part of metabolism**	**Total no. of metabolic reactions network (no. of unique)**	**No. of unique reactions common to other metabolic networks**	
		
		***A. niger***^1^	***S. cerevisiae***
**Biochemical reactions**	**1095 (681**^1^**)**	**191**	**580**

C-compound metabolism	463 (220)	157	160
Energy metabolism	20 (17)	11	13
Aminoacid metabolism	238 (171)	3	170
Nucleotide metabolism	144 (114)	17	114
Lipid metabolism	175 (122)	1	115
Secondary metabolism	42 (25)	1	3
Nitrogen and sulphur metabolism	8 (7)	-	4

Polymerization, assembly and maintenance	5 (5)	1	1

**Transport processes**	**118 (113)**	**60**	**59**

**TOTAL**	**1213 (794)**	**251**	**639**

The number of unique reactions in the metabolic network of *A. nidulans *is shown in parenthesis. The metabolic functions may be or not assigned to ORFs.

^1 ^Note that the metabolic network for *A. niger *is only detailed for the metabolism of C-compounds (the other parts of the metabolism are represented by lumped reactions) [21], which justifies the relatively low number of reactions in common to both networks in what concerns the other parts of the metabolism.

^2 ^6 non-enzymatic steps are included.

Table 2 presents the number of biochemical reactions involved in each part of the metabolism considered in the analysis, as well as the number of transport processes. In addition, Table 2 shows the number of unique reactions and transport processes predicted to participate in the different parts of the metabolism of *A. nidulans *that are common to the metabolic networks of *A. niger *and *S. cerevisiae*.

### Evaluation of functional assignments

The reliability of the functional assignments was evaluated according to the criteria described in Materials and Methods, and the candidate ORFs were classified into categories, as shown in Additional file 1. The definition of these criteria, in particular of the cut-off values in the E-values of BLAST searches, was found to strongly determine the number of candidate ORFs to consider for each metabolic function, and hence the reactions to include in the metabolic network for *A. nidulans*. The lists of the ORFs considered for each of the metabolic functions, for different stringencies of the criteria (i.e. cut-off in E-values) is presented in Additional file 2, whereas the data presented in Additional file 1 refer to a cut-off E-values of 1E-50.

### Functions with no ORF associated

After employing the algorithm described above, some of the metabolic functions predicted to occur in *A. nidulans *still remained without a link to a specific ORF in the genome. These metabolic functions corresponded to biochemical conversions for which no ORFs (hits) were identified by homology-based comparative analysis, or to those candidate ORFs that were subsequently neglected for not complying with the criteria considered (see Results – Evaluation of functional assignments). Nevertheless, 33 of these metabolic functions (or biochemical reactions) were considered to be part of the metabolic network, since they were essential for growth (see Results – Essential genes).

### Metabolic model

The metabolic reconstruction served as a basis to develop a mathematical model that describes the stoichiometry of all the metabolic processes in *A. nidulans*. The model comprised 1213 metabolic reactions, of which 1095 were biochemical transformations and 118 were transport processes. In addition, the model included 732 metabolites that could be balanced, i.e. metabolites whose net rate of their formation could be balanced with their net rate of consumption. Out of the 1213 reactions, there were 794 unique reactions (681 unique biochemical conversions and 113 unique transport processes), i.e. 419 of the reactions in the metabolic network were redundant. All the reactions in the metabolic network are listed in Additional file 1, along with a list of the abbreviations considered for the metabolite names (Additional file 2). Compartmentation was considered and the allocation of the biochemical conversions and metabolites to the different intracellular compartments (cytosol, mitochondria, and glyoxysomes) was based on the metabolic models for *A. niger *and *S. cerevisiae*. Besides catabolic and biosynthetic pathways, the model also included polymerization reactions and a reaction describing the formation of biomass, which was considered as a drain of building blocks or macromolecules in appropriate ratios to produce 1 mmol of monomers in the macromolecule or 1 g dry-weight (DW), respectively. These ratios were calculated based on the composition of the macromolecules in terms of building blocks (taken from *A. oryzae *[30]) and on the composition of biomass in terms of macromolecules (taken from *A. nidulans *[31] for lipids and *A. oryzae *[30] for the remaining macromolecules). A reaction representing the consumption of ATP for non-growth associated purposes was also included in the metabolic model. The ATP costs in the polymerization of amino acids and nucleotides were predicted based on reports for *P. chrysogenum *[32]. Furthermore, the ATP requirement for the assembly of macromolecules (19 mmol ATP/g DW) and for maintenance (2.85 mmol ATP/(g DW.h)) were estimated based on experimental biomass yields of *A. nidulans *grown in glucose-limited chemostat cultures for different dilution rates [33]. The values calculated were comparable with those reported for *A. niger *and *P. chrysogenum *[21].

### Model predictions

#### Essential genes

Single gene deletions were simulated and the capability of the corresponding mutants to grow on several carbon sources was determined (minimal media, no supplements). Table 3 shows the number of metabolic functions that were predicted to be essential for growth of *A. nidulans *on each of the four different substrates studied (glucose, xylose, glycerol, and ethanol), along with a list of the essential genes. The results from these studies were used to further refine the metabolic model. In fact, these investigations provided evidence for the existence of some metabolic steps for which no assignments were made based on homology searches. Therefore, these "missing functions" were considered to be part of the metabolic network for the sake of having an operative metabolic model.

**Table 3:** Essential ORFs in *A. nidulans *for growth on any of the four carbon sources investigated.

**Part of the metabolism**^1^	**No. of essential functions assigned to ORFs (total no.)**	**Essential ORFs**^2^
**BIOCHEMICAL REACTIONS**		

C-compound metabolism		
Tricarboxylic Acid Cycle	1 (1)	AN2999.2
One-carbon metabolism	2 (2)	AN1524.2, AN2998.2
Folate biosynthesis	2 (8)	AN8188.2, AN6032.2
Coenzyme A and pantothenate biosynthesis	4 (10)	AN1778.2, AN2526.2, AN0205.2, AN9446.2
Glycerol metabolism	1 (1)	AN1396.2
C6 metabolism	2 (3)	AN5975.2, AN2867.2
Chitin biosynthesis	3 (4)	AN5794.2, AN4234.2, AN9094.2
Glycogen biosynthesis	1 (1)	AN8010.2

**Amino acid metabolism**		
Arginine metabolism	6 (6)	AN7722.2, AN8770.2, AN1150.2, AN4409.2, AN1883.2, AN2914.2
Cysteine metabolism	1 (1)	AN2229.2
Glutamate and glutamine metabolism	1 (1)	AN4159.2
Glycine, serine and threonine metabolism	3 (3)	AN8859.2, AN4793.2, AN2882.2
Histidine metabolism	6 (6)	AN3748.2, AN2293.2, AN6536.2, AN0717.2, AN7044.2, AN7430.2
Branched chain amino acid metabolism	7 (4)^3^	AN4323.2/AN7878.2/AN5957.2, AN4956.2/AN4430.2, AN6346.2, AN0840.2
Lysine metabolism	4 (4)	AN8519.2, AN5610.2, AN5601.2, AN2873.2
Methionine metabolism	4 (4)	AN1263.2, AN4443.2, AN8277.2, AN1222.2
Aromatic amino acids metabolism	16 (17)^3^	AN0708.2/AN8886.2/AN4350.2, AN5731.2, AN6866.2, AN6338.2, AN5959.2, AN3695.2, AN3634.2, AN0648.2, AN6231.2/AN5444.2, AN4577.2, AN5200.2, AN1689.2, AN0648.2
Proline metabolism	1 (1)	AN7387.2

**Nucleotide metabolism**		
Purine metabolism	11 (11)	AN1395.2, AN6637.2, AN6541.2, AN5922.2, AN8121.2, AN3626.2, AN4739.2, AN4464.2, AN0893.2, AN5716.2, AN5566.2
Pyrimidine metabolism	9 (9)	AN0961.2, AN5909.2, AN5884.2, AN6157.2, AN4258.2, AN8213.2, AN3581.2, AN7028.2, AN0490.2
Salvage pathways	1 (1)	AN8216.2

**Lipid metabolism**		
Fatty acids metabolism	3 (1)^3^	AN5904.2/AN9408.2/AN9407.2
Phospholipids metabolism	12 (10)^3^	AN5599.2, AN6139.2, AN5166.2, AN5661.2, AN2154.2, AN1376.2, AN2261.2, AN6610.2, AN6580.2, AN6712.2/AN6211.2/AN7604.2
Sterol metabolism	12 (12)	AN4923.2, AN3869.2, AN2311.2, AN4414.2, AN0579.2, AN8012.2, AN3376.2, AN7751.2, AN5585.2, AN7146.2, AN0451.2, AN4042.2
Glycerolipid metabolism	1 (1)	AN6159.2
Glycolipids metabolism	0 (2)	-

**Sulfur metabolism**	1 (1)	AN1752.2

**TRANSPORT PROCESSES**	**0 (11)**	**-**

**TOTAL**	**115 (136)**	

Number and list of essential ORFs in *A. nidulans *for growth on any of the four carbon sources investigated, namely glucose, xylose, glycerol, and ethanol. The total number of metabolic reactions that are essential for growth, with or without an ORF associated is shown in parenthesis.

^1 ^Some ORFs encode enzymes that participate in different parts of the metabolism, but these are represented only once in the table.

^2 ^This list corresponds to all essential genes for growth on glucose, which also revealed to be essential for growth on any of the other carbon sources. However, additional genes were predicted to be essential on xylose (AN6037.2), glycerol (AN6037.2), and ethanol (AN6037.2, AN2916.2/AN2332.2/AN8793.2, AN8707.2, AN6653.2).

^3 ^In these cases, the number of essential ORFs may be greater than the number of essential biochemical conversions due to the presence of enzyme complexes.

#### Biomass yields

**Figure 1:**
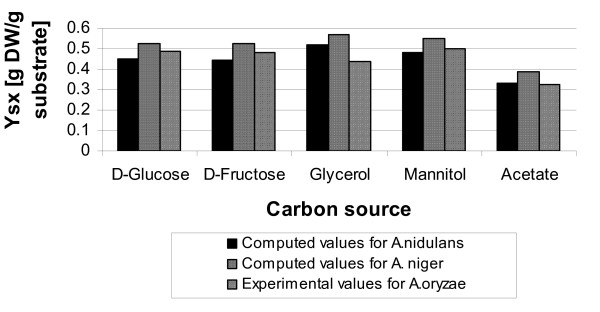
**Maximum theoretical and experimental growth yields for *Aspergillus***. Maximum theoretical growth yields predicted for *A. nidulans *(this study) and *A. niger *[21], and experimentally observed yields for *A. oryzae *[34].

The model was simulated to predict the maximum theoretical growth yields of *A. nidulans *on different carbon sources. Fig. 1 shows a comparison of these values with the maximum theoretical growth yields predicted for *A. niger *[21] and experimentally observed yields for *A. oryzae *in carbon-limited chemostat cultures [34]. All computations were performed considering the experimental substrate uptake rates in order to account for the relative effect of substrate consumption for maintenance purposes.

### Integration of large-scale expression analysis data

The reconstructed metabolic network was used in combination with data from large-scale transcriptional studies conducted with *A. nidulans*, in order to detect overall metabolic responses to specific genetic and environmental perturbations, using an algorithm developed by Patil and Nielsen [27]. This algorithm integrates gene expression data with topological information from metabolic models and enables the identification of small and coordinated changes in expression levels due to genetic or environmental perturbations. By using this algorithm, it is possible to identify *highly regulated or reporter metabolites *(i.e. metabolites around which the most significant changes in transcription occur) and *highly correlated subnetworks *(i.e. sets of connected genes with significant and coordinated transcriptional response to a perturbation), which enable to uncover metabolic responses to perturbations from transcriptional profiles.

The expression data sets used in this study represented the transcription levels of approximately one third of all predicted ORFs in *A. nidulans *(i.e. 3,278 ORFs) and concerned a reference strain grown on media containing different carbon sources (either glucose or ethanol), as well as a mutant strain that was impaired in the carbon repression system due to deletion of the carbon catabolite repressor gene *creA *[28]. Transcription analysis was therefore carried out for triplicates of four different cultivation conditions, namely the reference strain grown on glucose, the reference strain grown on ethanol, the *creA *mutant strain grown on glucose, and the *creA *mutant strain grown on ethanol. Differentially expressed genes were identified by using a two-way ANOVA, resulting in the calculation of two p-values for each ORF: a p-value concerning the effect of the growth medium, which was independent of the genotype of the strain, and a p-value concerning the effect of the genotype, which was independent of the medium used. Of the 3,278 ORFs included in the expression data sets, 571 ORFs were represented in the metabolic model developed in this study and thus the corresponding transcription profiles were considered for the analysis of reporter metabolites and metabolic subnetworks. The top 30 reporter metabolites, ranked according to p-values concerning the effect of the medium and genotype, are listed in Table 4. It was observed that, out of the top 30 reporter metabolites identified for each category (i.e. differential expression according to medium or genotype), only 8 metabolites were common to both categories, i.e. genes connected to these metabolites were regulated as both a result of the carbon source and the mutation. Moreover, 4 out of these 8 metabolites were involved in amino acid biosynthesis (namely N-Acetyl-L-glutamate 5-phosphate (mitochondrial), L-Tyrosine, N-(L-Arginino)succinate, N-Acetyl-L-glutamate 5-semialdehyde (mitochondrial)), and the highest ranked reporter metabolites in each category were involved in the biosyntheses of the amino-acids arginine and threonine.

**Table 4:** Top 30 reporter metabolites.

**Reporter metabolites**	
**Effect of growth medium**	**Effect of genotype**

N-Acetyl-L-glutamate (mitochondrial)	4-Phospho-L-aspartate
Methanol	*L-Xylulose*
Ethanol	*D-Sorbitol*
Ethanol (mitochondrial)	N-Acetyl-D-glucosamine 1-phosphate
Acetaldehyde (mitochondrial)	Oxaloglutarate (mitochondrial)
Acetaldehyde	*Acetate (mitochondrial)*
Carnitine	Glutathione
O-Acetylcarnitine	NH3 (extracellular)
*N-Acetyl-L-glutamate 5-phosphate (mitochondrial)*	*3-(4-Hydroxyphenyl)pyruvate*
D-Lactate	*L-Tyrosine*
Acetyl-CoA (mitochondrial)	D-Xylulose
Formaldehyde	*N-(L-Arginino)succinate*
*3-(4-Hydroxyphenyl)pyruvate*	O-Phospho-L-homoserine
*L-Tyrosine*	D-Mannitol
*L-Xylulose*	L-Glutamate
*D-Sorbitol*	*N-Acetyl-L-glutamate 5-phosphate (mitochondrial)*
CoA (mitochondrial)	Acetate
*N-(L-Arginino)succinate*	Starch (extracellular)
*Acetate (mitochondrial)*	Glycogen (extracellular)
2-Hydroxybutane-1,2,4-tricarboxylate (mitochondrial)	*N-Acetyl-L-glutamate 5-semialdehyde (mitochondrial)*
*N-Acetyl-L-glutamate 5-semialdehyde (mitochondrial)*	Glycerol 3-phosphate
Tartrate	NH3
Oxaloglycolate	gamma-Amino-gamma-cyanobutanoate
NAD+ (mitochondrial)	Sterigmatocystin
NADH (mitochondrial)	Dihydrosterigmatocystin
4-Aminobutyraldehyde (mitochondrial)	Versiconal hemiacetal acetate
4-Aminobutanoate (mitochondrial)	Water (mitochondrial)
(S)-Lactaldehyde (mitochondrial)	Xylitol
L-Kynurenine	D-Arabitol
L-Ornithine (mitochondrial)	N6-(1,2-Dicarboxyethyl)-AMP

Reporter metabolites identified based on the reconstructed metabolic network and expression data [28]. Two lists are shown, which correspond to highly regulated metabolites (ranked according to p-values) upon a change in the growth medium and in the genotype. Common metabolites to both lists are shown in italic.

Methanol, ethanol, and acetaldehyde were also found as very highly ranked reporter metabolites in the list concerning the effect of the medium, which is an indication of the differential regulation of a whole set of dehydrogenases, and is also suggested by the emergence of NAD^+ ^and NADH as reporter metabolites. Moreover, L-xylulose and D-sorbitol, which are involved in the metabolism of polyols, were ranked second and third, respectively, in the list of reporter metabolites concerning the effect of the genotype. Furthermore, it was observed that metabolites participating in the formation of secondary metabolites (e.g. sterigmatocystin) were among the 30 most highly regulated metabolites.

In order to obtain a more general overview of the effect of changing environmental/genetic conditions on the metabolism, the results obtained were subjected to further analysis. For the purpose, we made use of the links between the metabolic models available for *A. nidulans *and *S. cerevisiae*, which enabled to transfer specific tools for the analysis of -omics data in yeast to *A. nidulans*. Thus, all the enzymes associated to the top 30 reporter metabolites (listed in Table 4) were identified through the metabolic reconstruction developed for *A. nidulans*. Based on the EC numbers of these enzymes, the corresponding ORFs in *S. cerevisiae *were retrieved via a genome-scale metabolic model previously developed for yeast [22]. The "GO" terms associated to the yeast ORFs were then used to further study the metabolic effects of changing the carbon source and the genotype, using the "GO term finder" available at the *Saccharomyces *Genome Database [35] (see Table 5). The analysis of GO terms showed that, even though changes in the carbon source and genotype were reflected in common parts of the metabolism (e.g. carboxylic acid and organic acid metabolism), there were also clear differences between the effects exerted by the two categories. The change of carbon source seemed to affect mainly the energy metabolism of the cells, whereas a mutation that disturbs the carbon repression mechanism seemed to have a significant impact on the amino acid metabolism.

**Table 5:** Analysis of reporter metabolites and neighboring enzymes, using the "GO term finder".

**Effect of growth medium p-values for different media**		**Effect of genotype p-values for different strains**	
**GO term**	**Probability**	**GO term**	**Probability**

Carboxylic acid metabolism	1.73E-15	Carboxylic acid metabolism	5.24E-16
Organic acid metabolism	1.73E-15	Organic acid metabolism	5.24E-16
Generation of precursor metabolites and energy	1.89E-13	Amino acid biosynthesis	1.13E-13
Energy derivation by oxidation of organic compounds	1.11E-12	Amine biosynthesis	2.80E-13
Cellular metabolism	1.55E-09	Amine metabolism	6.05E-13
Amino acid biosynthesis	1.86E-09	Amino acid metabolism	1.84E-12
Metabolism	2.46E-09	Amino acid and derivative metabolism	4.67E-12
Fermentation	3.25E-09	Cellular metabolism	5.27E-09
Amine biosynthesis	3.59E-09	Metabolism	8.56E-09
Main pathways of carbohydrate metabolism	5.19E-09	Acetate metabolism	1.99E-08
Nonprotein amino acid biosynthesis	6.82E-09	Aspartate family amino acid metabolism	2.06E-08
Tricarboxylic acid cycle intermediate metabolism	7.64E-09	Nitrogen compound biosynthesis	8.25E-08
Acetate metabolism	1.26E-08	Arginine biosynthesis	8.25E-08
Nonprotein amino acid metabolism	3.42E-08	Cellular biosynthesis	9.29E-08
Coenzyme metabolism	1.32E-07	Biosynthesis	2.65E-07
Amino acid metabolism	1.47E-07	Nitrogen compound metabolism	3.19E-07
Malate metabolism	1.81E-07	Urea cycle intermediate metabolism	4.12E-07
Amino acid and derivative metabolism	2.70E-07	Arginine metabolism	4.12E-07
Aldehyde metabolism	5.36E-07	Glutamine family amino acid metabolism	6.78E-07
Amine metabolism	5.84E-07	Nonprotein amino acid biosynthesis	2.01E-06

Analysis of reporter metabolites and neighboring enzymes, using the "GO term finder" available at the *Saccharomyces *Genome Database [35] and the corresponding ORFs in yeast, retrieved via the reconstructed metabolism of S. cerevisiae [22].

## Discussion

### Metabolic reconstruction

According to the *Aspergillus nidulans *Database [5], only 194 of the metabolic ORFs included in the metabolic model developed for *A. nidulans *had been assigned a function in the metabolism before this study, and hence there was a large potential for functional annotation of metabolic genes. This potential was demonstrated by the functional assignment of 472 orphan ORFs. Yet, a number of issues arose in the course of the annotation of the metabolic genes in *A. nidulans*.

One of these issues was related to limitations inherent to a pathway-driven gene finding approach. In particular, the use of metabolic networks from selected organisms as references does not allow the identification of metabolic functions that are not present in these templates. In this way, additional or alternative reactions or pathways to these templates that may make part of the metabolic network of *A. nidulans *will not be predicted to exist, and subsequently the corresponding genes will not be identified using this methodology. In order to minimize this, the metabolic networks used as reactions databases in this work concerned organisms closely related to *A. nidulans*, and thus the major part of the metabolic reactions present in this fungus were likely to be covered.

Another issue is that our identification of candidate ORFs encoding a given function relied on comparative analysis, based on sequence similarity between the proteins in *A. nidulans *and other organisms. However, there probably exist enzymes in *A. nidulans *that are encoded by genes with low similarity to those encoding the same function in other organisms. Hence, queries based on BLAST searches will not result in identification of such genes. This problem was partially overcome by using, as queries, proteins of phylogenetically related organisms, for which many genes are likely to be conserved.

As alternatives to analytical tools relying on sequence similarity, there are tools based on genome context. However, gene clustering on the chromosome and analysis of shared regulatory sites as well as motif profiling are mainly relevant for comparative genomics in prokaryotes [16].

The selection of ORFs among the candidates encoding each function involved the specification of certain criteria and the development of a classification system. Hereby the reliability of the assignment of specific metabolic functions to candidate ORFs could be evaluated objectively and systematically, and furthermore it was possible to classify each annotation into categories according to the criteria defined. These criteria were essentially based on the E-values of BLAST searches and on the consistency between the function of interest and the function of the BLAST hits. All ORFs that were classified into at least one of the categories defined (A/A*, F, Y/Y* and O/O*, see Material and Methods – Evaluation of functional assignments) were considered in the metabolic model, whereas the ORFs that did not fall into any of the categories were not incorporated in the model. The categories A*, Y*, and O* were included to take into account also those ORFs for which the results from the comparative analysis were not conclusive, but did not contradict the function in question (e.g. aldose reductase *versus *xylose reductase; mitochondrial isocitrate dehydrogenase *versus *cytosolic isocitrate dehydrogenase). In this way, we aimed at identifying all ORFs that were associated to a given function (which might also represent isoenzymes or subunits of enzyme complexes), even though, by not being so stringent, we may have run into the problem of also finding false positives. Furthermore, assignment of ORFs to these categories may be valuable in future annotation studies.

The choice of the cut-off E-value in the BLAST searches is obviously determining the quality of our annotation. A number of factors influence the E-values in BLAST searches, such as the length of the queries and the size of the databases. Hence, the candidate ORFs were ranked according to the score to length ratio, as it hereby was possible to compensate for the fact that smaller proteins lead to higher E-values (smaller scores) in BLAST searches, because the probability of finding them by chance in the database is higher. Moreover, in order to have an idea on the order of magnitude of reasonable E-values for the different BLAST searches, previously annotated ORFs were used as positive controls. However, universal criteria may not apply, since the proteins' sequences may have diverse properties in the different parts of the metabolism (size, content, etc). Therefore, a sensitivity analysis was performed, in which different cut-off values in the E-values were considered in the BLAST searches. The stringency of this criterion determined the number of metabolic functions assigned to ORFs, as well as the number of ORFs considered for each function (see Additional file 3). A cut-off of 1E-50 in the E-values was found to be reasonable and hence this value was chosen for all BLAST searches in the selection of candidate ORFs and development of the metabolic model.

On the other hand, the differences in the order of magnitude of the E-values in the different BLASTP searches carried out can explain the fact that some ORFs were not classified into the category O (i.e., have an homolog, different from the query sequence, in the NCBI protein database [26]). It would be expected that all candidate ORFs would fall into this category, since these were found using as queries, protein sequences retrieved from this database. However, by setting the same cut-off in the E-values for all BLASTP searches, we may have been too stringent in some cases, namely when searching homologs for the candidate ORFs in the protein database at NCBI, which is a very large database and hence the probability of finding the query sequence by chance is high, resulting in high E-values.

As mentioned above, all BLAST hits for a given function whose E-values were below the cut-off and that were classified into at least one of the categories described were considered to encode that function. However, in some cases, the discrimination between ORFs encoding isoenzymes and subunits of enzyme complexes was difficult, particularly for those cases in which there was no information available for *A. nidulans *or this could not be directly extrapolated from other organisms. For example, even though in *S. cerevisiae *there are two genes (YDR341C and YHR091C) encoding the enzyme arginyl tRNA synthetase (EC 6.1.1.19), only a single ORF (AN6368.2) was identified in *A. nidulans*, through BLASTP searches. Similarly, a single ORF was found in *A. nidulans *(AN5610.2) for L-aminoadipate-semialdehyde dehydrogenase (EC 1.2.1.31), which is a complex enzyme in yeast containing two subunits (a large and a small subunit), encoded by two different genes (YBR115C and YGL154C, respectively).

This study therefore shows clearly that the outcome of comparative genomics is highly dependent on the criteria chosen, and also highlights the importance of the application of a systematic evaluation to the obtained annotations.

### Analysis of expression data using the metabolic network topology

Transcriptomics has become a cornerstone of functional genomics over the last decade and the analysis and interpretation of microarray data has proven to be a new challenge. Even for well-studied model organisms, the analysis and interpretation of transcriptome data is often hampered by the numerous genes that have no function assigned. This becomes even more evident when transcriptomics moves on to genomes whose annotation barely relies on automated gene prediction. In these cases, basically any additional information can be very helpful for the analysis and interpretation of data. As shown previously by our group, the metabolic network reconstructed in this work can be valuable for upgrading the information content in transcriptome data [29], i.e. to provide a link between gene expression profiles and integrated metabolic functions. The importance of the reconstructed metabolic network in the analysis of transcriptome data of *A. nidulans *was again illustrated in this work with a study concerning transcriptional responses to changes in the growth medium composition (glucose and ethanol) of a wild type strain and a mutant strain with deletion of the regulatory protein CreA [28]. The initial transcription data set used comprised 3,278 ORFs, of which 571 ORFs had an assigned function in the metabolic network. Although the selected expression data subset (consisting of 571 ORFs) did not cover the whole metabolic network reconstructed for *A. nidulans*, the reporter metabolites identified after applying the method from Patil and Nielsen [27], still provided valuable information on the underlying metabolic changes, which reflects the robustness of the method.

For both categories (differential expression according to growth medium or genotype), the highest ranked reporter metabolites were involved in the biosynthesis of amino acids (arginine and threonine, respectively) (Table 4), which was not expected, since it was anticipated that the different environmental/genetic conditions would mainly affect the carbon metabolism. In particular for the transcriptional responses due to changes in the carbon source, the involvement of the amino acid metabolism was further reflected in many of the other reporter metabolites (Table 4).

On the other hand, this approach may play an important role in functional genomics, by giving insight into the perturbations that lead to specific transcriptional responses (for example, in the analysis of strains that are mutated in genes with unknown function). In fact, from the list of reporter metabolites presented in Table 4, it is possible to deduce that major changes occurred in the metabolism of carbon compounds in *A. nidulans*, in particular in the metabolism of ethanol (from reporter metabolites, such as ethanol, acetaldehyde, acetate, acetyl-CoA, etc.). However, tracing back the underlying perturbations from the topology of the network and observed transcriptional responses is still a challenging task, even for well-studied organisms.

Furthermore, the reconstructed metabolic network for *A. nidulans *allows the utilization of tools that have been developed by the yeast community for the analysis of -omics data. This represents an advantage, because one can rely on the linkage of the expression data to a specific reaction rather than to a specific gene. After identification of the reporter metabolites and the reactions in which they participate, the reconstructed metabolic network of *S. cerevisiae *can be used to identify ORFs in yeast that are involved in the same reactions and use these for further analysis. As an example, the "GO term finder" available at *Saccharomyces *Genome Database was used to identify specific parts of the metabolism related to the reporter metabolites. The results (reporter metabolites) obtained based on the differential expression according to the medium revealed many GO categories that could be directly linked to the change of the carbon source from glucose to ethanol, and therefore again showed that by using this approach one can obtain a birds-eye view on the major metabolic changes. The results based on the changes in the genotype of the strains, i.e. deletion of the carbon repression mediator CreA, showed that the highest ranked reporter metabolite, as well as other lower ranking ones, was involved in the amino acid biosynthesis, which was also confirmed by the GO term analysis. This linkage between glucose repression and amino acid biosynthesis was further substantiated by the finding that a defined set of genes that is regulated via CreA also has binding sites for CpcA (homologue of GNC4 from *S. cerevisiae*) that is a major regulator of the amino acid metabolism [28]. This shows that apparently the CreA protein is not only involved in the mediation of carbon repression, but plays an even more global role in the metabolism of the cell. The emergence of polyols in the list of reporter metabolites confirms previously reported results [36,37], and the expression data therefore allow tracking down the origin of these changes in the metabolism.

## Conclusion

In this work, we illustrated the use of a pathway-driven approach to improve the functional annotation of the genome of *A. nidulans*. Moreover, we showed how the metabolic reconstruction establishes functional links between genes, enabling the upgrade of the information content of transcriptome data.

## Methods

**Figure 2:**
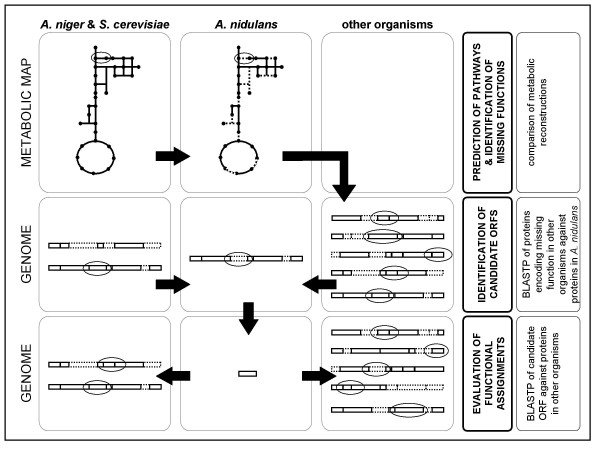
Diagram depicting the pathway-driven approach to functional annotation of ORFs adopted in this work.

**Figure 3:**
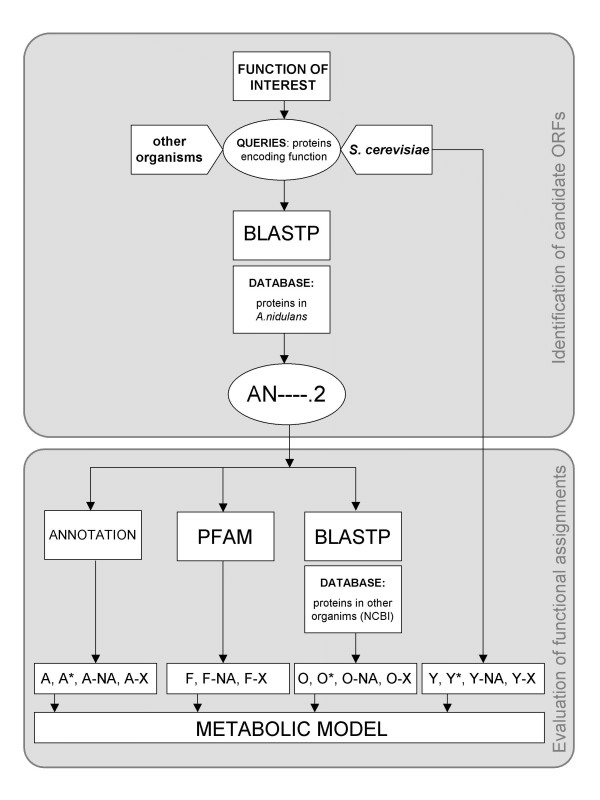
Diagram representing the steps in the annotation process.

The approach employed in this work for the annotation of the metabolic genes within the genome of *A. nidulans *was based on the method previously described by Osterman and Overbeek [16]. The different steps carried out are depicted in Fig. 2 and 3, and described in the following.

### Metabolic reconstruction

The central metabolism of *A. nidulans*, as well as selected pathways from its secondary metabolism, was reconstructed using as templates detailed metabolic reconstructions of other organisms. For the reconstruction of the metabolism of carbon compounds in *A. nidulans*, a detailed metabolic reconstruction for *A. niger *[21] was used as reaction database, whereas the metabolisms of amino acids, nucleotides, and lipids were predicted based on reactions identified in *S. cerevisiae *[22], for which a more comprehensive reconstructed metabolic network was available. As the energy metabolism of *S. cerevisiae *is simpler than for many other eukaryotic cells, the energy metabolism of *A. nidulans *was reconstructed using as references the corresponding pathways from both yeast [22] and mouse [23]. The biosynthetic routes of secondary metabolites, namely penicillin and aflatoxins, were based on detailed studies of these pathways in *P. chrysogenum *[24] and in different species of *Aspergillus *[25], respectively. Evidence for the presence of these pathways in *A. nidulans *was in part supported by available genomic data, such as previously annotated ORFs [5], sequenced and cloned genes (with or without an ORF associated) [38], Expressed Sequence Tags (ESTs) [39] and Tentative Consensus sequences (TCs) [40]. The metabolic pathways in *A. nidulans *were also predicted based on biochemical evidence given by reports on isolation and characterization of enzymes in this fungus [41,42]. Furthermore, the inclusion of some reactions in the metabolic reconstruction of *A. nidulans *was supported solely by physiological evidence (e.g. the ability to grow on a certain sugar as the sole carbon and energy sources require a transporter and a complete pathway for metabolism of this sugar).

The directionality and reversibility of the reactions included in the metabolic network of *A. nidulans *were based on those in the metabolic networks of other organisms that served as templates for the reconstruction.

### Identification of candidate ORFs

Once the metabolic network of *A. nidulans *was reconstructed and the missing functional roles identified, the genome of *A. nidulans *was surveyed for the genes encoding the corresponding enzymes, by employing comparative genomics tools based on sequence similarity. This approach was also applied for the identification of ORFs encoding metabolic functions that had been previously assigned to genes in *A. nidulans*, aiming at identifying isogenes. The sequences of a set of proteins with the designated enzymatic activity in other organisms (queries) were compared with the set of all predicted proteins in *A. nidulans *[5]. The protein sequences used as queries were obtained from the (non-redundant) NCBI protein database [26] (searched by the corresponding enzyme commission numbers whenever possible), and represented well-characterized enzymes in other organisms, preferentially in organisms taxonomically related to *A. nidulans *(e.g. fungi or eukaryota). BLASTP (blosum62) was used for comparative analysis and assigning metabolic functions to ORFs in the genome of *A. nidulans*. The selection of the best candidate ORFs for a given function relied on the cut-off in the expectation values (E-values) considered, as well as on the score-to-sequence length ratio. Protein sequences or translated nucleotide sequences were used in the comparisons, rather than nucleotide sequences, due to the presence of introns, which characterizes eukaryota, and thus filamentous fungi. For the cases in which no hits were generated in this way, the translated nucleotide sequences of genes encoding the specific enzymes (obtained from the (non-redundant) NCBI nucleotides database [26]) were used as queries, and BLASTX was employed for generating putative assignments.

### Evaluation of functional assignments

Once candidate ORFs were identified for the metabolic functions, the reliability of the functional assignments was assessed and the ORFs were classified into different categories, according to several criteria, as shown in Fig. 3 and Table 6, and described in the following.

**Table 6:** Classification of candidate ORFs into categories, according to several criteria.

**Functional annotation (A)**	
*Comparison of the previous functional annotations for the candidate ORFs [5] with the function of interest*	
**A**	function assigned to the candidate ORF consistent with function of interest
A*	function assigned to the candidate ORF not contradicting function of interest (e.g. aldose reductase *versus *xylose reductase; mitochondrial isocitrate dehydrogenase *versus *cytosolic isocitrate dehydrogenase)
**A-NA**	no function assigned to the candidate ORF (e.g. hypothetical protein)
**A-X**	function assigned to the candidate ORF not consistent with function of interest

**Protein families (F)**	

*Comparison of the protein domains originally predicted for the candidate ORF [5] with those of the enzymes catalysing the function of interest [6]*	
**F**	protein family of candidate ORF consistent with protein family of function of interest
**F-NA**	no protein family associated to the candidate ORF
**F-X**	protein family of candidate ORF not consistent with protein family of function of interest

**Comparative genomics with yeast (Y)**	

*BLASTP searches of all proteins in S. cerevisiae [35] against all predicted proteins in A. nidulans [5]*	
**Y**	function of the protein in yeast (that has a match with the candidate ORF) consistent with missing function
Y*	function of the protein in yeast (that has a match with the candidate ORF) not contradicting missing function (e.g. aldose reductase *versus *xylose reductase; mitochondrial isocitrate dehydrogenase *versus *cytosolic isocitrate dehydrogenase)
**Y-NA**	no match of protein in yeast with the candidate ORF
**Y-X**	function of the protein in yeast (that has a match with the candidate ORF) not consistent with missing function

**Comparative genomics with other organisms (O)**	

*BLASTP searches of candidate ORF in A. nidulans against (non-redundant) protein database at NCBI [26]*	
**O**	functions of proteins in other organisms (that have a match with the candidate ORF) consistent with missing function
O*	functions of proteins in other organisms (that have a match with the candidate ORF) not contradicting missing function (e.g. aldose reductase *versus *xylose reductase; mitochondrial isocitrate dehydrogenase *versus *cytosolic isocitrate dehydrogenase)
**O-NA**	no matches of candidate ORF with proteins in other organisms
**O-X**	functions of proteins in other organisms (that have a match with the candidate ORF) not consistent with missing function

#### Functional annotation (A)

Previous annotations of the candidate ORFs (if available in the *Aspergillus nidulans *Database [5] or in The *Aspergillus nidulans *Linkage Map [38]) were compared with the function in question. The candidate ORFs were classified into the categories A, A*, A-X or A-NA, depending on the availability and consistency of the results of the comparison (see Fig. 3 and Table 6).

#### Protein families (F)

The protein domains originally predicted for the candidate ORFs [5] were compared with those of the enzymes catalyzing the functions of interest [6], and the candidate ORFs were classified accordingly into the categories F, F-X or F-NA (see Fig. 3 and Table 6).

#### Comparative genomics with yeast (Y)

The proteins in *S. cerevisiae *[35] were used as queries in BLASTP searches against all predicted proteins in *A. nidulans *[5], and the correspondences between ORFs in this yeast and *A. nidulans *generated in this way were used to evaluate the reliability of the candidate ORFs. This was accomplished by comparing the functions assigned to the ORFs in yeast with those assigned to the candidate ORFs. The candidate ORFs were then classified into the following classes Y, Y*, Y-X or Y-NA (see Fig. 3 and Table 6).

#### Comparative genomics with other organisms (O)

Potential homologs of the candidate ORFs encoding the metabolic functions in *A. nidulans *were surveyed in other organisms, through BLASTP searches of the former against the (non-redundant) protein database at NCBI [26]. The corresponding functions were compared and the candidate ORFs were classified into the categories O, O*, O-X or O-NA (see Fig. 3 and Table 6).

### Isoenzymes, multifunctional enzymes and subunits in enzyme complexes

The BLAST searches often yielded several hits (or candidate ORFs) with E-values lower than the cut-off value (or with similar score-to-length ratios) for a given function. These hits could potentially correspond to ORFs encoding isoenzymes or subunits of enzyme complexes. In these situations, all candidate ORFs were considered and classified according to the criteria described above. In some cases, the existence of these hypothetical isoenzymes or subunits in *A. nidulans *was supported by information concerning this fungus available in the literature. Otherwise, information was extrapolated from *A. niger *or *S. cerevisiae*.

Multi-enzyme complexes and isoenzymes were represented in a different way in the metabolic reconstruction. All subunits considered to belong to a same multi-enzyme complex were associated to a single reaction in the metabolic network, whereas isoenzymes were represented as independent reactions. For example, in the metabolic network of A. nidulans, the pyruvate dehydrogenase complex is represented by two subunits (AN5162.2/AN9403.2), which are associated to a single reaction (EC 1.2.4.1). On the other hand, two isoenzymes of glutamate dehydrogenase (AN7451.2 and AN4376.2) were associated to two different reactions in the metabolic network, a NAD^+^- and a NADP^+^-dependent reaction, respectively. Reactions catalysed by multi-enzyme complexes were included, if at least one of the subunits was identified.

Another issue that was addressed was related to the assignment of different functions to the same ORF. These ORFs could possibly encode multifunctional proteins, and hence these were surveyed for the existence of multiple protein domains to verify the hypotheses.

### Metabolic model and simulation methods

The reconstructed metabolic network of *A. nidulans*, including reactions encoded by previously annotated genes as well as metabolic functions initially missing, served as a basis to develop a stoichiometric model. The model was subsequently used for simulating microbial growth and for gene deletion analysis, by employing flux balance analysis and linear programming methods [21,43].

### Application of the reconstructed metabolic network for the analysis of transcription data

The reconstructed metabolic network was used in combination with a computational method published by Patil and Nielsen [27] for analysis of large-scale gene expression data referring to a study on glucose repression in *A. nidulans*. This method enables the identification of so-called reporter metabolites and metabolic subnetworks, based on their interconnectedness within the metabolic network through common metabolites and on information about changes in the expression level of the genes.

This approach was applied to analyze expression data concerning a reference strain and a *creA *deleted strain of *A. nidulans*, grown on different carbon sources, specifically glucose and ethanol [34]. Furthermore, the top 30 reporter metabolites identified based on the changes in gene expression between the different carbon sources (glucose *versus *ethanol) or genotype of the strains (reference *versus creA *deletion mutant) were used in combination with information on the topology of the metabolic network of *S. cerevisiae *[22] for further analysis, using tools available at the *Saccharomyces *Genome Database [35].

## Authors' contributions

HD performed the annotation work and drafted the manuscript. ISO carried out the annotation work. GH participated in the design of the study and helped to draft the manuscript. JN participated in the design and coordination of the study and helped to draft the manuscript. All authors read and approved the final manuscript.

## Supplementary Material

Additional file 1 **List of the reactions comprising the metabolic model developed for *A. nidulans***. List of the reactions comprising the metabolic model developed for *A. nidulans*. Each reaction is associated to an ORF within the genome of *A. nidulans *(when available), and to the EC number of the corresponding enzyme (when available). The classification of the ORFs into categories is also presented. The functional assignments were based on a cut-off in the E-values of BLAST searches of 1E-50. The reactions that are common to the metabolic networks of *A. nidulans *and *S. cerevisiae *are represented by AN and SC, respectively. Click here for file

Additional file 2 **List of metabolites (abbreviations and full names) included in the metabolic model for *A. nidulans***. In the abbreviations, the suffixes 'm', 'g', 'e' stand for metabolites localized in the mitochondria, glyoxysomes and extracellular medium, respectively. No suffix was added to cytosolic metabolites. Click here for file

Additional file 3 List of the ORFs associated to each biochemical reaction for different cut-off values in the E-value of BLASTP searches Click here for file
